# Liver Cancer Cell Lines Treated with Doxorubicin under Normoxia and Hypoxia: Cell Viability and Oncologic Protein Profile

**DOI:** 10.3390/cancers11071024

**Published:** 2019-07-20

**Authors:** Ilse R. Dubbelboer, Natasa Pavlovic, Femke Heindryckx, Erik Sjögren, Hans Lennernäs

**Affiliations:** 1 Department of Pharmacy, Uppsala University, Box 580, 751 23 Uppsala, Sweden; 2 Department of Medical Cell Biology, Uppsala University, Box 571, 751 23 Uppsala, Sweden

**Keywords:** IC_50_, doxorubicin, liver cancer, cell lines, hypoxia, normoxia

## Abstract

Hepatocellular carcinoma is often treated with a combination of doxorubicin and embolization, exposing it to high concentrations and hypoxia. Separation of the possible synergistic effect of this combination in vivo is difficult. Here, treatment with doxorubicin, under hypoxia or normoxia in different liver cancer cell lines, was evaluated. Liver cancer cells HepG2, Huh7, and SNU449 were exposed to doxorubicin, hypoxia, or doxorubicin + hypoxia with different duration. Treatment response was evaluated with cell viability, apoptosis, oxidative stress, and summarized with IC_50_. The protein profile of a 92-biomarker panel was analyzed on cells treated with 0 or 0.1 µM doxorubicin during 6 or 72 h, under normoxia or hypoxia. Hypoxia decreased viability of HepG2 and SNU499. HepG2 was least and SNU449 most tolerant to doxorubicin treatment. Cytotoxicity of doxorubicin increased over time in HepG2 and Huh7. The combination of doxorubicin + hypoxia affected the cells differently. Normalized protein expression was lower for HepG2 than Huh7 and SNU449. Hierarchical clustering separated HepG2 from Huh7 and SNU449. These three commonly used cell lines have critically different responses to chemotherapy and hypoxia, which was reflected in their different protein expression profile. These different responses suggest that tumors can respond differently to the combination of local chemotherapy and embolization.

## 1. Introduction

Intermediate hepatocellular carcinoma (HCC) is treated with image-guided transcatheter tumor therapy by locally infusing a chemotherapeutic agent in a drug delivery system, so called transarterial chemoembolization (TACE) [1,2,3,4]. This treatment has two purposes: to deliver the drug close to the tumor site and to cause an arterial embolization close to the cancer region [5]. The embolization reduces or eliminates the blood flow to the tumor, reducing the tumor’s oxygen supply and creating a hypoxic microenvironment. By infusing the drug close to the tumor (locoregional), the tumor is exposed to a high concentration of the drug, which will lead to an increased antitumor effect, while reducing systemic exposure and side-effects [6]. One of the most commonly used chemotherapeutic agents of this locoregional treatment of HCC is doxorubicin (DOX) [7], an anthracycline which has been on the market since the 1970s. Its main mechanisms of action lead to the activation of apoptotic pathways and are intercalation into DNA, inhibition of topoisomerase II, and generation of reactive oxygen species [8,9,10]. DOX has been used for the treatment of advanced HCC for over 30 years, but definitive evidence that DOX as a systemic treatment improves survival has not been provided [11], while the survival benefit in combination with chemoembolization has been generally accepted [12].

Tumor hypoxia is a key hallmark of all solid tumors, including HCC. An inevitable consequence of chemoembolization therapy is the de novo formation of hypoxic regions within the tumor [13]. Hypoxic cancer cells undergo phenotypic adaptations that allow the cells to survive or escape from the hostile environment, often creating a more aggressive tumor phenotype [14]. These hypoxia-induced adaptations include switching to anaerobic metabolism, inducing angiogenesis, stimulating tissue remodeling, and activating molecular mechanisms that allow invasion and metastasis [15,16]. Apparently, hypoxia may act as a double-edged sword when it comes to drug-response. On the one hand, intratumoral hypoxia and the hypoxic phenotype have been implicated in resistance to radiotherapy and anticancer chemotherapy, as well as predisposing for increased tumor metastases [17]. On the other hand, depriving the cells of their oxygen supply—for instance by inhibiting neoangiogenesis—has been a successful strategy for extending the survival of patients. This is currently the standard of care for patients with advanced HCC [18]. The efficacy of the cytostatic agent sorafenib in the advanced setting suggest that antiangiogenic therapy may enhance the efficacy of chemoembolization, yet this combinational treatment has generated contradictory results when it comes to overall survival [19,20,21]. Also, preclinical studies report conflicting results on whether intratumoral hypoxia enhances or reduces efficacy of chemotherapeutic agents [22,23].

Human cancer-derived cell lines are the most widely used in vitro models to study cancer mechanistically and to validate the efficacy of cancer treatments [24]. One of the main advantages of using cell lines is that they offer a nearly infinite supply of a homogeneous cell population. However, this homogeneity is only found within the same cell line and can also be seen as a disadvantage considering the heterogeneity of in vivo tumors [5]. Thus, a panel of different cancer cell lines, representative of the natural heterogeneity observed in primary tumors, could be used instead [25].

In this study, the objective was to investigate the tolerance to DOX and/or hypoxic treatment of three commonly used liver cancer cell lines [25,26,27]. HepG2 cells are derived from a 15-year old Caucasian American male with a well-differentiated liver tumor [28]. Huh-7 cells, a differentiated HCC cell line taken from a liver tumor in a 57-year-old Japanese male [28]. SNU449 cells is a hepatitis B-positive cell line derived from a 52-year-old Korean male with a primary HCC [28]. The cells were treated with different DOX concentrations at different exposure times and under normoxic or hypoxic conditions. The effect of the treatments was assessed by their antitumor effect (cell viability and cell death), presence of oxidative stress, and the change in expression in a number of cancer proteins that participate in biological mechanisms central to the initiation and progression of cancer [29].

## 2. Results

### 2.1. Cell Viability—Treatment with DOX under Hypoxic or Normoxic Conditions

**Figure 1:**
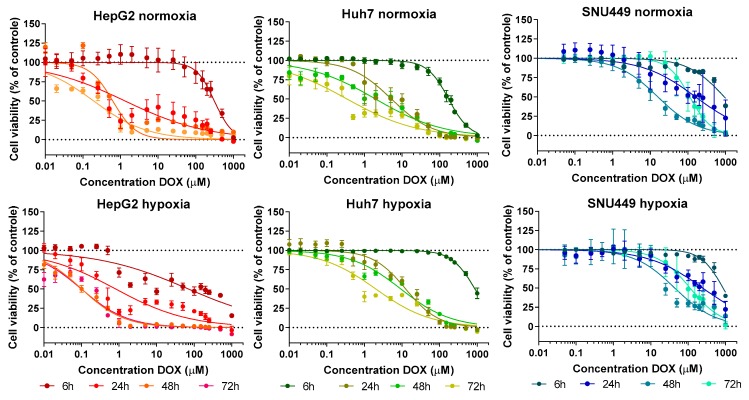
The effect of different treatments on cell viability over time. Doxorubicin exposure–response curves for the three cell lines (left to right: HepG2, Huh7, and SNU449) at the different exposure times. Cells were kept under normoxia (top) or chemical hypoxia with 100 µM CoCl_2_ (bottom) during the duration of the experiment. Average (symbols) and standard deviation (error bars) are shown for each exposure of DOX concentration; at least 6 replicates were used for each tested concentration.

Effect of hypoxia on cell viability over time is summarized in Table 1. Presence of hypoxia after exposure to 100 µM CoCl_2_ was confirmed by measurement of HIF1α and PDK1 after CoCl_2_ treatment confirmed presence of hypoxia (Supplementary Material, Figure S1). The effect of treatments with DOX and/or hypoxia on cell viability can be seen in Figure 1, and the corresponding IC_50_ values are shown in Table 2.

Cell viability of HepG2 cells grown under hypoxic conditions declined to 81% from 6 to 72 h compared to normoxic conditions (Table 1). Tolerance (IC_50_) of HepG2 cells to DOX decreased 1600-fold from 6 to 72 h of exposure in normoxic conditions (Table 2; Figure 1). Tolerance to DOX decreased between 1.6- and 6-fold at each time point when HepG2 were exposed to DOX under hypoxic conditions (Table 2; Figure 1).

Under hypoxic conditions, Huh7 cell viability was unaffected or even slightly increased compared to normoxic conditions (Table 1). Similar to the HepG2 cells, tolerance of Huh7 cells to DOX decreased in normoxic conditions, here with 500-fold from 6 to 72 h of exposure (Table 2; Figure 1). Tolerance of Huh7 to DOX increased between 2- and 6-fold at each time point when exposed to DOX under hypoxic conditions (Table 2; Figure 1).

SNU449 cell viability declined to 76% from 6 to 48 h in hypoxic conditions compared to normoxia, but cell viability recovered to baseline at 72 h (Table 1). Under normoxic conditions, tolerance of SNU449 to DOX first declined ~60-fold over time (48 h), and then increased at 72 h to an 8-fold decline of IC_50,6h_ (Table 2; Figure 1). Tolerance to DOX of SNU449 cells under chemical hypoxia was only slightly affected (<3-fold) compared to normoxic conditions (Table 2; Figure 1).

### 2.2. Oxidative Stress and Apoptosis

**Figure 2:**
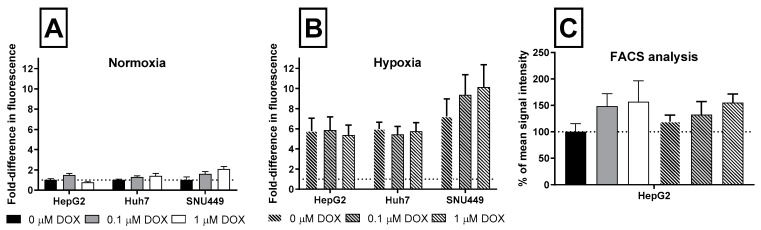
Effects on the oxidative stress cells experience with different DOX concentrations. Panel A and B show the oxidative stress to cells under normoxia and chemical hypoxia using a DCFDA—Cellular Reactive Oxygen Species (ROS) Detection Assay Kit in the microplate format. Panel C shows the same experiment, except that DCFDA was measured by flow cytometry. Results are shown as mean fold difference (**A**&**B**) or % of mean signal intensity (**C**) of control condition (normoxia and 0 µM DOX), error bars show SD. Six replicates were used for each tested condition.

**Figure 3:**
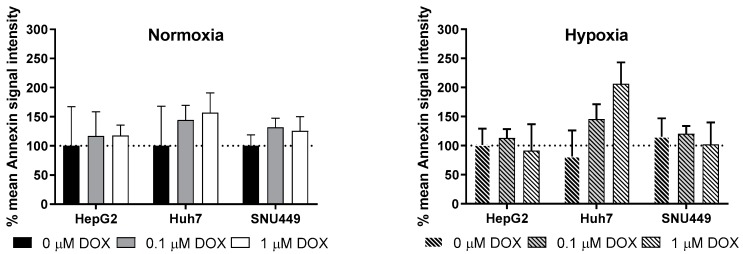
The apoptotic response of cells exposed to different concentrations of DOX for 24 h. Results are shown as percentage of mean Annexin V signal intensity of control conditions (normoxia and 0 µM DOX), with SD as error bars. Three replicates were used for each tested condition.

The effect of treatment with DOX after 24 h on oxidative stress and apoptosis are shown in Figure 2 and Figure 3. Under normoxia, 0.1 µM DOX led to a nonsignificant increase of oxidative stress in all cell lines after 24 h (Figure 2A). A higher exposure of 1 µM DOX lead to a nonsignificant increase of oxidative stress in Huh7 and SNU449, but decreased oxidative stress levels in HepG2 cells (Figure 2A). Since this method does not normalize for total cell number, we also measured DCFDA using flow cytometry and selecting living cells. This revealed a significant increase of oxidative stress in HepG2 cells treated with 0.1 and 1 µM DOX (Figure 2C). Oxidative stress was significantly increased in all cells exposed to CoCl_2_-induced hypoxia (Figure 2B). Interestingly, only the SNU449 experienced a significant additive effect of hypoxia and DOX on oxidative stress levels (Figure 2B).

HepG2 cells showed no change in the apoptotic marker Annexin V during any of the treatments. Under normoxia, levels of Annexin-V nonsignificantly increased in the Huh7 and SNU449 cells treated with 0.1 and 1 µM for 24 h (Figure 3). Huh7 cells showed a nonsignificant increase of Annexin V, after 24 h exposure of 0.1 µM and 1 µM DOX in hypoxic conditions. Under hypoxia, the SNU449 cells had no increased levels of the apoptotic marker Annexin-V after treatment with DOX.

### 2.3. Oncologic Protein Profile

**Figure 4:**
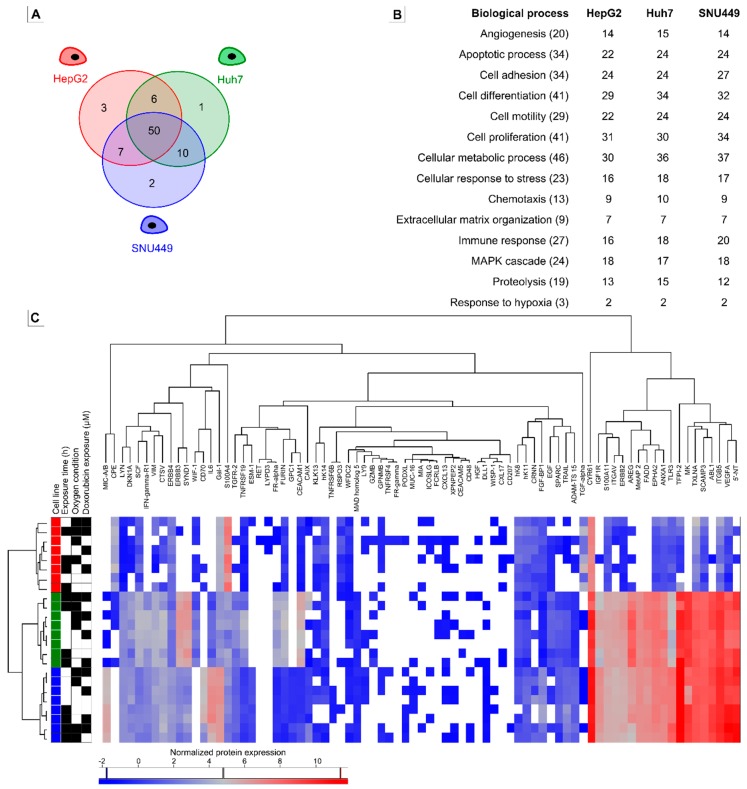
Results of oncological protein profiling. (**A**) Venn diagram showing the number of detected proteins in each cell line. (**B**) The number of detected proteins for each biological process in each cell line. For each biological process, the number of proteins included in the analysis are given in brackets behind the process. Note that proteins can have several biological process classes. (**C**) Hierarchical clustering based on proteomic quantification visualized in a heat map. A two-way unsupervised hierarchical clustering of the median protein expression values of all proteins in three cell lines—HepG2 (red), Huh7 (green), and SNU449 (blue)—treated during 6 (white) or 72 h (black) under chemical hypoxia (black) or normoxia (white) and exposed to no doxorubicin (white) or 0.1 µM doxorubicin (black). The colored bar under the heat map represents the abundance of the different proteins in the heat map; blue is low abundance, red is high abundance. White squares in the heat map show NA values, i.e., not detected in at least 2 of 3 samples. Raw data is shown in Supplementary Material, Figure S2.

Of the 92 proteins included in the OLINK Oncology panel [29], 79 proteins (84.9%) were detected in at least one median sample (Figure 4A). The majority (50) of proteins were detected in all three cell lines, while six proteins were detected in one cell line only (Supplementary Material, Figure S2). Sixty-six proteins (71.7%) were detected in at least one median sample of the HepG2, 67 (72.8%) in Huh7, and 69 (75%) in SNU449 cells. Significant differences in protein expression were observed between HepG2 and Huh7 for 37 proteins (Table 3). The normalized expression of 35 proteins was significantly different between HepG2 and SNU449, and normalized expression of 32 proteins was significantly different between Huh7 and SNU449 (Table 3). Changes in exposure time, oxygen conditions, or DOX exposure did not result in significantly different protein expressions (results not shown). The number of detected proteins per biological process class (GO terms) per cell line are shown in Figure 4B.

Hierarchical clustering of samples (Figure 4C, left side heat map) revealed that the type of cell line was the most important factor contributing to the protein profile. Neither exposure time, nor oxygen conditions or DOX exposure caused any further subclustering. Hierarchical clustering of expressed proteins is shown Figure 4C (top side). Protein clustering could not be assigned to any biological process, molecular function, or cellular component (GO terms; results not shown).

The heatmap (Figure 4C) shows the different normalized protein expression of each protein in each sample. For a number of detected proteins, HepG2 cell samples had lower expression of proteins compared to Huh7 and SNU449 cell samples (Figure 4C, right side heat map, Table 3). In HepG2 cells only eight proteins had a normalized protein expression higher than 2 (average from all HepG2 samples, irrespective of treatment), while 34 proteins in Huh7 cells and 35 proteins in SNU449 cells were detected with normalized protein expression over 2.

## 3. Discussion

The cytotoxic potency of DOX and the synergistic effects of chemical hypoxia on cell viability, oxidative stress, and cell death were investigated for three liver cancer cell lines (HepG2, Huh7, and SNU449). These cell lines were selected based on previously published data on their response to DOX and their morphological features.

The observed tolerance to DOX was significantly different between the cell lines, where HepG2 cells exhibited the lowest and SNU449 cells the highest tolerance. For example, at 72 h exposure to DOX there was a 580-fold difference between IC_50_ values for HepG2 and SNU449. Huh7 and HepG2 are epithelial cell lines, characterized by a high expression of E-cadherin and low expression of Vimentin [30]. In contrast, SNU449 has a mesenchymal phenotype, characterized by loss of E-cadherin and low expression of vimentin [30,31]. The study by Zhang, et al. in 2018 has shown that epithelial–mesenchymal transition is the driving factor behind DOX resistance in SNU449 cell lines [30,31]. In similarity to this study, Chang et al. in 2013 reported that tolerance to DOX is lower in HepG2 compared to SNU449 cell lines [32]. However, the Huh7 cell line was reported to be equally sensitive as HepG2 to DOX in the study by Chang et al. in 2013, while higher tolerance to DOX in Huh7 was observed in our study. Furthermore, there are large discrepancies in studies reporting on of DOX tolerance of the different cell lines. Reports on the sensitivity of HepG2 and Huh7 range from equivalent tolerance [33,34] up to 30-fold differences [32,35]. Also, reported IC_50_ values of DOX from different studies vary vastly. For example, after 48 h DOX exposure on HepG2 cells IC_50_ values varied from 0.038 to 1.9 µM [34,36,37]. This large interstudy variability indicates that it is not possible to compare DOX cytotoxicity between different studies and emphasize the need to include multiple cell lines for preclinical drug development studies. To enable cell-dependent comparisons of DOX cytotoxicity, experiments need to be performed within one study and cannot be solely based on compilations of external data. In general, reported in vitro IC_50_ values for DOX in human carcinoma cell lines decrease with exposure time [37,38]. This correlates well to the strong in vivo PK–PD relationship for DOX treatment observed between plasma exposure (area under the plasma concentration–time curve (AUC)) and cell survival [8]. A 20-fold decrease in DOX IC_50_ is reported for HepG2 cells when increasing exposure time from 6 to 48 h, and similar effects (30-fold decrease) are observed in a ovarian cancer cell line when increasing exposure time from 2 to 12 h. [37,38] Our results are in line with these observations as DOX IC_50_ decreased between 60- and 500-fold, depending on cell line and increase in exposure time. This suggests that our cell lines were highly responsive to DOX. Interestingly, the SNU449 cell line showed higher tolerance at 72 h than after 48 h, having possibly built up resistance to DOX treatment. Several mechanisms have been described to contribute to the acquired resistance to DOX [39]. Firstly, an upregulation of multidrug resistance efflux pumps could create a reduction in nuclear drug accumulation [40]. Unfortunately, the biomarkers analyzed with the oncology panel did not represent proteins for drug response, which could be why there were no noticeable differences in hierarchical clustering on treatment levels. Secondly, reduction in DNA Topoisomerase 2-Alpha (TOP2A) expression and increased dependence on the beta-isoform of topoisomerase II contribute to the acquired resistance of DOX [39,41]. This beta isoform is less sensitive to DOX and would thus result in a decrease in the number of double-strand breaks and subsequent decrease in apoptosis. Thirdly, a downregulation of proapoptotic and upregulation of antiapoptotic proteins could also contribute to the acquired drug resistance in cells [39]. In our biomarker analysis, both VEGFA and MK expression in SNU449 were seemingly higher after 72 h cell incubation compared to 6 h. These proteins may cause induction of cell proliferation (MK) and inhibition of apoptosis (VEGFA) [42], which could result in the above-described resistance observed at 72 h. Lastly, the inherent differences in cell proliferation could have contributed to the different response of the cell lines to DOX. The tolerance of cells to DOX is known to be lower in proliferating cells than in nonproliferating cells [43]. An in vitro study on breast cancers cells has shown that tumor proliferation rate is one of the major biologic parameters associated with response to DOX [44]. SNU449 cells were the least sensitive to DOX treatment and they also have a slower doubling time compared to Huh7 and HepG2.

The difference in viability of cells after treatment with DOX is also reflected in their different expression of the apoptotic marker Annexin V. Under normoxia, Annexin-V increased in the Huh7 and SNU449 cells treated with 0.1 and 1 µM DOX for 24 h. This is in line with our viability data, which show a decrease of 3% in cell viability of Huh7 cells after 24 h of exposure to 0.1 µM and 29% after 24 h of exposure to 1 µM DOX in normoxic conditions. In contrast, the SNU449 cells had no increased levels of Annexin V, after treatment with DOX in hypoxic conditions. This is in line with our viability data, which show that SNU449 cells experience no decrease in cell viability at 0.1 µM and 1 µM DOX. Under hypoxia, Huh7 cells showed a significant increase of Annexin V, after 24 h exposure to 0.1 µM and 1 µM DOX. This partially corresponds to our viability data, which show no decrease in cell viability of Huh7 cells after 24 h of exposure to 0.1 µM, but a 15% after 24 h of exposure to 1 µM DOX in hypoxic conditions. Remarkably, HepG2 cells showed no change in the apoptotic marker Annexin V, suggesting other mechanism may be responsible for the decreased viability. Despite the widespread clinical use of DOX, its antiproliferative and death-inducing signal cascades are not yet fully understood. Studies have suggested that decreased viability seen after DOX treatment is a result of apoptosis, necrosis, cell cycle arrest, and senescence [45,46,47], which explains some of the discrepancies we see between expression of Annexin V and the viability data. Our data also suggest that these mechanisms can differ between different cell lines and further warrant the use of multiple cell lines for preclinical studies on HCC.

Inducing hypoxia with CoCl_2_ resulted in varying responses of the three cell lines. HepG2 and SNU449 cell viability declined when exposed to hypoxia. Huh7 had unaffected or slightly increased cell viability in hypoxic conditions, suggesting that Huh7 has a higher tolerance to hypoxia. It is important to note that HIF1α activity varies between different cancer cell lines under the same level of hypoxia [48]. The different levels of HIF1α and PDK1 found in our three cell lines, could perhaps explain differences in their adaptation to survive in hypoxic conditions. Notably, SNU449 cells had the highest protein level of stabilized HIF1α and PDK1 and its viability was decreased at 24 h and 48 h CoCl_2_-induced hypoxia, but recovered to normal levels at 72 h. This suggests that these cells adapted to the hypoxic conditions over time. In contrast, Huh7 has a higher tolerance to hypoxia, but also lower baseline levels of stabilized HIF1α and PDK1 in normoxic conditions. In agreement to our findings, a study on six human breast cancer cell lines found high basal levels of VEGF A and low HIF-1α and HIF-2α induction was correlated with improved survival under hypoxia [49]. HIF-1α is activated during hypoxia, here induced by CoCl_2_, and then activates transcription of both VEGF A and CAIX [42]. In our protein profile results, VEGF A was detected in all three cell lines at 6 and 72 h. Normalized protein expression of VEGF A was only slightly increased in SNU449 cells for all samples incubated 72 h, but more interestingly basal levels of VEGF A were 8-fold higher in Huh7 and SNU449 than in HepG2. Cell viability of both Huh7 and SNU449 was unaffected at 6 and 72 h in hypoxia, which is in agreement with the higher basal VEGF A expression. CAIX, a protein that helps with neutralizing intracellular pH and acidifying extracellular microenvironment [42,50] was detected in Huh7 and SNU449. CAIX was increased 8–fold in Huh7 cells treated for 72 h with hypoxia and 2-fold in SNU449 cells. This suggests that Huh7 and SNU449 cells actively adapt to the hypoxic conditions, which could have led to the sustained cell viability from 6 to 72 h.

Tolerance of cancer cell lines to chemotherapeutic agents can increase or decrease in hypoxic conditions. Increased tolerance to chemotherapeutics under hypoxic conditions are observed in human embryonal carcinoma testicular germ cell tumor cell lines [51], HCC cell lines (HepG2, BEL-7402, and SMMC-7721) [52,53], and human and mouse prostatic adenocarcinoma cells [54]. However, conflicting results have also been published, where hypoxia could cause either increased or decreased tolerance to different chemotherapeutic agents [55]. This is similar to the results in this study, where different effects of hypoxia on DOX tolerance were observed for the three cell lines included. For HepG2 cultured in hypoxia, the cell viability with DOX treatment decreased over time compared to non-DOX treated cells. On the contrary, cell viability for Huh7 cells was not affected over time under hypoxia, while the tolerance to DOX increased compared to normoxic conditions. This suggested that hypoxia induces a more DOX-resistant phenotype in these cells. Interestingly, the tolerance to DOX of SNU449 was only marginally affected by hypoxic conditions.

It has been hypothesized that cells that proliferate less in hypoxia will be more tolerant to chemotherapeutic agents, as the chemotherapeutic agents act on dividing cells [55]. DOX has three working mechanisms: topoisomerase inhibition, intercalation to DNA, and formation of reactive oxygen species [8,9,10]. The latter mechanism is often described in the context of adverse effects, such as cardiotoxicity, but also occurs in DOX-treated cancer cells [56]. Since CoCl_2_ does not create an actual oxygen deprivation, but mimics hypoxia by binding to prolyl hydroxylase containing domain proteins that activate HIF-1α, there is still an abundance of reactive oxygen species, which might have contributed to the DOX cytotoxicity [57]. In line with previous findings [58], we found an increase of oxidative stress in all cells exposed to CoCl_2_. A low exposure of 0.1 µM DOX induced oxidative stress in all cell lines after 24 h, which is in line with previous reports [56]. A higher exposure of 1 µM DOX also led to an increase of oxidative stress in Huh7 and SNU449, but decreased oxidative stress in HepG2 cells. Since this method did not normalize for total cell number, we assumed that this decrease could be a result of the high number of HepG2 cells dying because of the DOX treatment. Measuring ROS using flow cytometry and selecting living cells, indeed revealed an increase of oxidative stress in HepG2 cells exposed to 1 µM DOX. Interestingly, the tolerance to DOX of SNU449 was only marginally affected by hypoxic conditions, while SNU449 cells was the only cell line to increase the level of oxidative stress when CoCl_2_-induced hypoxia was combined with DOX treatment. This suggests that oxidative stress did not contribute to DOX cytotoxicity in the SNU499 cell line. Elevation of intracellular mitochondrial ROS levels in tumors is known to activate PKD1 and NF-κB, leading to upregulation antiapoptotic proteins such as A20 and cIAPs [59]. Therefore, generation of ROS in tumor cell can work as a double-edged sword, by both promoting and inducing cell death, depending on duration of oxidative stress, intracellular ROS levels, as well as cell-specific mechanisms that counter proapoptotic stimuli or prevent the activation of oxidative stress-induced signaling cascades [60].

DOX is known to upregulate and activate HIF-1α, and HIF-1α targeting strategies have been suggested to enhance the effect of DOX treatment [61]. Possibly, treatment with DOX could have led to a secondary activation of HIF-1α, which could induce a positive feedback loop to increase the number of prolyl hydroxylase containing domain proteins and thus interfere with the working mechanism of CoCl_2_ [62]. It is thus possible that the differences in tolerance to DOX under hypoxic conditions in the tested cell lines is caused by a different main antitumor effect for each cell line, or because of subjection to HIF-1α-induced feedback mechanisms that interfered with CoCl_2_. In our study, we found different levels of HIF1α and PDK1 in the three cell lines under the same oxygen conditions, which could explain differences in their adaptation to hypoxia and their sensitivity to DOX. In addition, there were differences in generation of ROS between the different cell lines under normoxia and hypoxia, before and after DOX treatment, which would further contribute to the cytotoxic effect of both hypoxia and DOX.

Another possible explanation of the different responses between cell lines, both in historical data and our results, could be the difference in how hypoxia was created. Chemical hypoxia with CoCl_2_ is a hypoxic mimetic that functions by stabilizing HIF-1α. It has been shown that the difference in oxygen levels during hypoxia could affect cell behavior, where induction of apoptosis was observed when oxygen levels decreased below 0.5%, but not at oxygen levels of 1–3% [63]. Interestingly, it was also found that tolerance to DOX both increased (1% O2) and decreased (0.1% O2) in five different cancer cell lines (not liver) [55], which is in agreement with the induction of apoptosis at lower oxygen levels.

A final important finding in our study was the difference in oncogenic protein profile between cell lines. Previous studies have shown a high similarity in gene expression between Huh7 and HepG2 cell lines and HCC tumor tissue, based upon rank-based gene expression [25,27]. The same study also observed a low similarity between the gene expression in SNU449 and HCC tumor tissue, and so discouraged readers to use SNU449 for in vitro experiments [25]. However, our results clearly show a higher degree of similarity between Huh7 and SNU449 cells compared to HepG2 cells. Most notable was the lower normalized expression of most oncogenic proteins in HepG2 cells compared to the other cell lines. The used oncologic protein panel is composed specifically of cancer proteins that participate in biological mechanisms central to the initiation and progression of cancer [29]. This could suggest that HepG2 cells could represent a less aggressive form of HCC tumors, compared to Huh7 and SNU449 cell lines. It is important to note that HepG2 cells are derived from a 15-year-old Caucasian American male and there is currently no real consensus whether these cells are derived from hepatocellular carcinoma [64] or from hepatoblastoma [65]. Irrespective of origin of HepG2, and in agreement with our results, Pang et al. in 2014 also concluded that HepG2 should not reflect the apoptotic and drug resistance properties of other HCC cell lines [26]. This discrepancy in historic tumor classifications, and our findings showing a differential oncogenic protein profile, raise questions on how representative this cell line truly is for research on initiation and progression of HCC.

## 4. Materials and Methods

### 4.1. Chemicals

Doxorubicin hydrochloride (DOX HCl) was purchased from Toronto Research Chemicals, Canada. DOX stock solutions (100 mM) were prepared by dissolving the DOX in DMSO (Sigma-Aldrich, Germany). RIPA buffer, resazurin sodium salt and phosphate buffered saline (PBS) were purchased from Sigma-Aldrich (Darmstadt, Germany). Dulbecco’s Modified Eagle’s Medium (DMEM), Roswell Park Memorial Institute medium (RPMI), fetal bovine serum (FBS), and trypsin-EDTA were purchased from Gibco. Cobalt (iii) chloride hexahydrate (CoCl_2_, Sigma-Aldrich, Darmstadt, Germany) stock solutions were prepared by dissolving CoCl_2_ in sterile water (25 mM) and filtering through a syringe filter (0.22 µm).

### 4.2. Cell Culture

**Figure 5:**
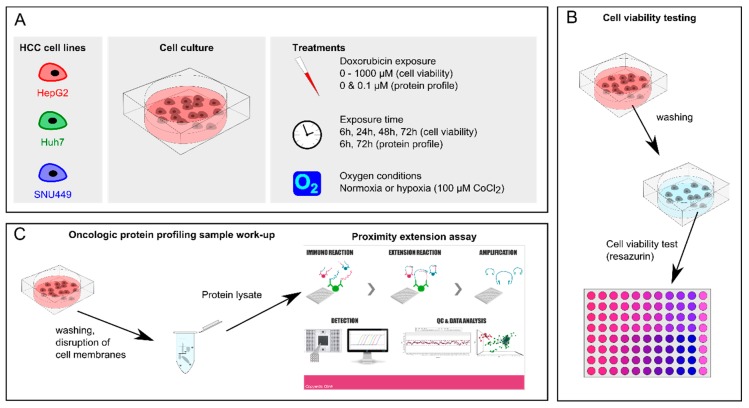
Overview of the used methods to assess the effect of treatments in hepatic cell lines. (**A**) The used liver cancer cell lines, cell culture in 96-well plates, and the applied treatments. (**B**) The sample work-up and analysis for cell viability testing. (**C**) Oncological protein profiling sample work-up and analysis by proximity extension assay [29].

Three HCC cell lines (Figure 5A) were selected based on published data concerning IC50 values of the most commonly used HCC and hepatoma cell lines (Table 4). Our literature search showed that the most drug-resistant tumor cell line commercially available, which mimics the gene expression pattern of the original HCC patient (in contrast to cell lines that were genetically engineered to be chemoresistant) was SNU449. HepG2 and Huh7 cells were chosen as described to be more sensitive to DOX and because of their common use in HCC research. Contamination of the three cell lines was checked at the Register of Misidentified Cell Lines, and none of the chosen cell lines were on the list [66]. The liver cancer cell lines HepG2 and SNU449 were purchased from ATCC and Huh7 was kindly provided by Ahmed Dilruba, Karolinska Institute. Cells were routinely cultured in DMEM (HepG2 and Huh7) or RPMI (SNU449) supplemented with 10% FBS (cell culture media+FBS: CCM_FBS_) and 1% antibiotic–antimycotic solution (Sigma-Aldrich, Darmstadt, Germany). Cells were cultured at 37 °C with 5% CO_2_ under standard cell culturing conditions (Figure 5A).

### 4.3. Cell Viability Assay

For in vitro experiments, cells were detached using trypsin-EDTA, re-suspended in CCM_FBS_, and plated at a density of 1 × 10^4^ cells/well. Cells were allowed to attach and left undisturbed to adhere overnight. Thereafter, cells were treated with monotherapies of DOX and hypoxia, respectively or as a combination DOX + hypoxia. DOX stock solution was serially diluted to concentrations in the range of 0.01 to 1000 µM in CCM_FBS_ (CCM_FBS_ + DOX, Figure 5A). For the chemically induced hypoxia experiments, CoCl_2_ stock solutions were diluted with cell culture medium (CCM_FBS_ + CoCl_2_) before making the 0, 0.01–1000 µM DOX concentration range in CCM_FBS_ + CoCl_2_ (CCM_FBS_ + CoCl_2_ + DOX, Figure 5A). A final concentration of 100 µM CoCl_2_ was used to induce chemical hypoxia, as previously described [70]. Medium was removed from the wells and 150 µL of CCM_FBS_ (+CoCl_2_) + DOX was added to each well. Plates were again incubated in a humidified incubator with 5% CO_2_ at 37°C for the desired exposure time (6, 24, 48, or 72 h), with and without chemically induced hypoxia (Figure 5A). Note that media was not replaced under the exposure time. Each treatment was tested on eight or more replicates. Medium was removed after the exposure time and cells were washed with 100 µL PBS (Figure 5B). Cell viability was monitored via resazurin reduction assay, which provides a cost-effective and accurate method to determine cell viability in cytotoxicity studies [71]. A 1% solution of resazurin sodium salt (Sigma-Aldrich) in PBS was filtered through a 0.22 µm filter. The filtered resazurin solution was added in 1/80 dilution to the cells and incubated for 24 h, after which fluorescent signal was measured with a 485/35 excitation filter and a 550/20 emission filter on a Fluostar Omega plate reader. The seeding density of the cells was confirmed to be within the limits of linearity between cell number and absorbance for this technique (Supplementary Material, Figure S3).

### 4.4. HIF_1_α and PDK_1_ in Cell ELISA Assay

Cells were seeded into _96_-well amine-coated plates at a density of 1 × 10^4^ cells/well and exposed to normoxic (CCM_FBS_) or hypoxic conditions (CCM_FBS_ + CoCl_2_, 100 µM) for 6, 24, and 72 h. HIF_1_α and PDK_1_ expression was measured using Hypoxic Response Human In-Cell ELISA Panel (Abcam, Cambridge, MA) according to manufacturer’s guidelines. Plates were scanned using an Odyssey imager (LI-COR, Lincoln, NE). Expression values were obtained by subtracting background values from wells with negative controls for each condition and normalized to the corresponding Janus Green fluorescence values for each well.

### 4.5. Oxidative Stress Measurement

Oxidative stress was measured using DCFDA—Cellular Reactive Oxygen Species (ROS) Detection Assay Kit (ab_113851_) in a microplate format and by flow cytometry. DCFDA is fluorogenic dye that measures ROS activity within the cell. After diffusion in to the cell, DCFDA is deacetylated by cellular esterases to a non-fluorescent compound, which is later oxidized by ROS into DCF, a highly fluorescent compound with maximum excitation and emission spectra of 495 nm and 529 nm respectively. Cells were seeded into 96-well plates with clear bottom and black sides at a density of 2.4 × 10^4^ cells/well for the microplate assay, and into 24-well plates at 1.2 × 10^5^ cells/well for flow cytometry and left to adhere overnight. On the next day, cells were stained with 25 µM DCFDA for 45 min at 37 °C, according to manufacturer’s guidelines. Following this step, cells were exposed to 0, 0.1 µM and 1 µM DOX in normoxic (CCM_FBS_) or hypoxic conditions (CCM_FBS_ + CoCl_2_, 100 µM). After 6 h of treatment, fluorescence was measured at 485 nm excitation and 535 nm emission wavelengths using a Fluostar Omega plate reader and BD FACSCallibur. Results of the microplate assay are shown as fold change fluorescence. Results of the flow cytometry measurements are shown as percentage of mean DCF signal intensity of control conditions.

### 4.6. Annexin V Flow Cytometry

Cells were seeded into 24-well plates at 1.2 × 10^5^ cells/well and left to adhere overnight. On the next day, cells were exposed to 0, 0.1 µM, and 1 µM DOX in normoxic (CCM_FBS_) or hypoxic conditions (CCM_FBS_ + CoCl_2_, 100 µM) for 24 h, following which apoptosis was assessed by flow cytometry using eBioscience™ Annexin V Apoptosis Detection Kit FITC (88-8005-72) according to manufacturer’s guidelines. Briefly, cells were washed with 1× Binding Buffer and incubated with a 1/20 dilution of fluorochrome-conjugated Annexin V for 10 min, after which fluorescence was measured at 485 nm excitation and 535 nm emission wavelengths using BD FACSCallibur. Results are shown as percentage of mean Annexin V signal intensity of normoxic untreated condition.

### 4.7. Oncologic Protein Profile

Preparation of the cells was as per the previous section. Cells were seeded in _6_-well plates at 2 × 10^5^ per well and left to adhere overnight. DOX stock solution was diluted to 0.1 µM in CCM_FBS_ (+ CoCl_2_). Eight different conditions were tested per cell line: normoxia or chemically induced hypoxia with exposure to 0 or 0.1 µM DOX during 6 or 72 h, with three replicates per experimental condition. Medium and cell debris were removed and remaining cells washed with PBS. Cells were detached with 100 µL ice-cold RIPA containing protease inhibitors (Sigma Aldrich), and cells were scraped off and aspired (Figure 5C). Cell suspension was collected in Eppendorf tubes and kept on ice for 20–30 min, whilst mixing vigorously to enhance disruption of the cell membranes. The cell suspensions were centrifuged (20 min, 13,000 rpm, 4 °C) and supernatant containing protein was collected. Supernatant was stored at −20 °C until protein measurement. Protein concentration was measured using the BCA kit (ThermoFisher). Finally, all supernatants were diluted to 1 mg/mL protein in RIPA. All samples were analyzed with a multiplex proximity extension assay for 92 biomarkers in the oncology panel (Olink Bioscience, Uppsala, Sweden) [29].

### 4.8. Data Analysis

Cell viability, defined as the percentage of fluorescence value of treated cells compared to fluorescence value of untreated cells, was calculated with Equation (1):

(1) Cell viability (%)=(Fluorescence exp well–Average of Fluorescence blank well)/(Average of Fluorescence control well–Average of Fluorescence blank well)

The blank wells were empty in the plate while control wells contained cells in medium without any additional DOX treatment. Cell viability data vs. DOX concentrations were plotted in GraphPad Prism (version 7.04, GraphPad Software Inc., San Diego, CA, USA) and fitted with the “inhibitor vs. response—variable slope analysis” to determine the _50_% inhibitory concentration (IC_50_) of DOX. DOX IC_50_ values were determined per cell line, for each exposure time, for both normoxic and hypoxic conditions. Differences between DOX IC_50_ with and without hypoxia per cell line was tested statistically with a _2_-way ANOVA and Tukey’s multiple comparisons test (significant when adjusted *p* < 0.05) in GraphPad Prism. The effect of hypoxia was assessed by taking the ratio of cell viability under hypoxic and normoxic conditions. The effect of hypoxia on cell viability was tested statistically with a one-sample *t*-test (difference from _1_) in GraphPad Prism.

Protein expression data from all different treatments on the three cell lines (Figure 5C) was obtained from OLINK [29], and processed using the following steps. (1) All data below LOQ or at LOQ were assumed to be not detected (NA). (2) All samples that had not passed the quality control were assumed to be not detected (NA). (3) Take median of duplicates or triplicates if at least two samples had a value over LOQ, otherwise median was set to NA. Analysis and visuals presented in this report are based on this processed data set. Hierarchical clustering was performed in Perseus (version 1.6.0.7) [72], where standard parameters were selected. Statistically differently expressed proteins between cell lines, exposure time, oxygen condition and DOX exposure was determined using volcano plots in Perseus.

## 5. Conclusions

In this study, we present our findings that three established and commonly used liver cancer cell lines (HepG_2_, Huh7, and SNU_449_) have critically different responses to chemotherapy and hypoxia. This was also reflected in their oncogenic protein profile and their response to hypoxia and oxidative stress. A synergic effect of hypoxia and treatment with DOX was only observed in HepG_2_ cells, while Huh7 and SNU_449_ might have developed escape mechanisms from both treatment and hypoxia. These escape mechanisms are of uttermost importance when studying chemotherapeutic agents. Adaptation of tumor cells to hypoxia is believed to be the main driver for selection of more invasive and therapy-resistant cancer phenotypes [73]. Our results emphasize the need to consider inter- and intratumoral heterogeneity and include multiple cell lines in preclinical studies. Our study further suggests that tumors can respond differently to the combination of local chemotherapy and embolization, which is important for future treatment optimization. This treatment is often a combination of the local intrahepatic administration of one or more chemotherapeutic agents, combined or followed with an occlusion of the tumor feeding vessels. There are two purposes of the treatment: obtaining high local chemotherapeutic tumor concentrations and creating a hypoxic environment to cause synergistic cell death. However, our findings suggest that this might not always be the best strategy. They support the numerous clinical findings that the combination of vessel occlusion and chemotherapeutic treatment does not cause increased overall survival [5].

## Figures and Tables

**Table 1:** The effect of exposure time of chemical hypoxia with 100 µM CoCl_2_ on cell viability of HepG2, Huh7, and SNU449 liver cancer cell lines. Ratio of hypoxia effect was calculated as cell viability at hypoxia/normoxia with 0 µM doxorubicin, and is described as average with standard deviation; at least 6 replicates were used for each tested concentration.

Time	HepG2	Huh7	SNU449
6 h	1.01 ± 0.045	1.01 ± 0.0055 *	0.99 ± 0.022
24 h	1.03 ± 0.14	1.04 ± 0.064	0.95 ± 0.077 *
48 h	0.90 ± 0.072 *	1.06 ± 0.0091 *	0.76 ± 0.082 *
72 h	0.81 ± 0.1 *	0.99 ± 0.009 *	1.02 ± 0.044

* indicate a significant difference (*p* < 0.05) from 1 as tested with one-sample *t*-test.

**Table 2:** IC_50_ values (µM) of DOX for cell lines at specified time intervals and oxygen conditions following exposure to the drug. Chemical hypoxia was induced with 100 µM CoCl_2_. IC_50_ values are given as mean ± standard error; at least 6 replicates were used for each tested concentration.

Time	HepG2			Huh7			SNU449		
	Normoxia	Hypoxia	R_hyp/norm_	Normoxia	Hypoxia	R_hyp/norm_	Normoxia	Hypoxia	R_hyp/norm_
**6 h**	310 ± 12	70 ± 11 *	0.2	170 ± 3.3 ^a^	870 ± 15 *^,a^	5.1	920 ± 72 ^a,b^	860 ± 32 ^a^	0.9
**24 h**	1.3 ± 0.18^c^	0.81 ± 0.087	0.6	5.2 ± 0.49 ^c^	12 ± 0.78 ^c^	2.4	160 ± 17 ^a,b,c^	240 ± 16 *^,a,b,c^	1.5
**48 h**	0.62 ± 0.06 ^c^	0.1 ± 0.0046	0.2	2 ± 0.18 ^a,c^	9.2 ± 0.58 ^c^	4.7	16 ± 0.73 ^c,d^	44 ± 3.7 ^c,d^	2.8
**72 h**	0.19 ± 0.017 ^c^	0.099 ± 0.0089	0.5	0.34 ± 0.038 ^c^	2 ± 0.17 ^c^	5.8	110 ± 3.9 ^a,b,c,e^	110 ± 5.5 ^a,b,c,d,e^	1.0

* statistically different from normoxia at same time point and cell line; ^a^ statistically different from HepG2 at same time point and oxygen condition; ^b^ statistically different from Huh7 at same time point and oxygen condition; ^c^ statistically different from same cell line and oxygen condition at 6 h; ^d^ statistically different from same cell line and oxygen condition at 24 h; ^e^ statistically different from same cell line and oxygen condition at 48 h. Tested with 2-way Anova with Tukey’s multiple comparisons test, significant when adjusted *p* < 0.05. R_hyp/norm_ ratio IC_50_ hypoxia/normoxia.

**Table 3:** Analyzed proteins and their biological process together with the difference in normalized protein expression between cell lines.

Protein	Biological Process ^†^	HepG2 vs. Huh7	HepG2 vs. SNU449	Huh7 vs. SNU449
5′-nucleotidase (5′-NT; P21589)	Cell adhesion; Cellular metabolic process	---- *	---- *	-- *
A disintegrin and metalloproteinase with thrombospondin motifs 15 (ADAM-TS 15; Q8TE58)		- *	-- *	-- *
Alpha-taxilin (TXLNA; P40222)	Cell proliferation; Cellular response to stress	--- *	--- *	-
Amphiregulin (AREG; P15514)	Cell differentiation; Cell proliferation; Cellular metabolic process	---- *	--- *	++ *
Annexin A1 (ANXA1; P04083)	Apoptotic process; Cell adhesion; Cell differentiation; Cell motility; Cell proliferation; Cellular metabolic process; Cellular response to stress; Chemotaxis; Immune response	--- *	--- *	+
Carbonic anhydrase 9 (CAIX; Q16790)	Cellular metabolic process; Cellular response to stress; Response to hypoxia	--	-- *	+
Carboxypeptidase E (CPE; P16870)	Cellular metabolic process; Proteolysis	+++ *	NA	NA
Carcinoembryonic antigen-related cell adhesion molecule 1 (CEACAM1; P13688)	Angiogenesis; Cell adhesion; Cell differentiation; Cell motility; Cell proliferation; Cellular metabolic process; Immune response; MAPK cascade	--- *	- *	+++ *
Carcinoembryonic antigen-related cell adhesion molecule 5 (CEACAM5; P06731)	Apoptotic process; Cell adhesion; Cell differentiation	NA	NA	NA
Cathepsin L2 (CTSV; O60911)	Cell differentiation; Cell proliferation; Cellular metabolic process; Cellular response to stress; Extracellular matrix organization; Proteolysis	-- *	-- *	+
CD160 antigen (CD160; O95971)	Cell proliferation; Immune response;	ND	ND	ND
CD27 antigen (CD27; P26842)	Apoptotic process; Cell differentiation; Cell proliferation; Cellular metabolic process; Cellular response to stress; Immune response; MAPK cascade	ND	ND	ND
CD48 antigen (CD48; P09326)	Cell motility	+	-	-
CD70 antigen (CD70; P32970)	Apoptotic process; Cell proliferation; Immune response	NA	--- *	NA
Cornulin (CRNN; Q9UBG3)	Cell adhesion	+	-	-
C-type lectin domain family 4 member K (CD207; Q9UJ71)		NA	-	NA
C-X-C motif chemokine 13 (CXCL13; O43927)	Angiogenesis; Cell adhesion; Cell motility; Cell proliferation; Chemotaxis; Immune response	-	-	+
Cyclin-dependent kinase inhibitor 1 (DKN1A; P38936)	Apoptotic process; Cell differentiation; Cell proliferation; Cellular metabolic process; Cellular response to stress	-- *	-- *	+
Delta-like protein 1 (DLL1; O00548)	Angiogenesis; Apoptotic process; Cell adhesion; Cell differentiation; Cell proliferation; Cellular metabolic process; Immune response	NA	NA	-
Disintegrin and metalloproteinase domain-containing protein 8 (ADAM 8; P78325)	Angiogenesis; Apoptotic process; Cell adhesion; Cell differentiation; Cell motility; Cellular metabolic process; Cellular response to stress; Chemotaxis; Extracellular matrix organization; Immune response; MAPK cascade; Proteolysis; Response to hypoxia	ND	ND	ND
Endothelial cell-specific molecule 1 (ESM-1; Q9NQ30)	Angiogenesis; Cell proliferation	NA	--	NA
Ephrin type-A receptor 2 (EPHA2; P29317)	Angiogenesis; Apoptotic process; Cell adhesion; Cell differentiation; Cell motility; Cell proliferation; Cellular metabolic process; Cellular response to stress; Chemotaxis; MAPK cascade	--- *	--- *	+ *
FAS-associated death domain protein (FADD; Q13158)	Apoptotic process; Cell adhesion; Cell differentiation; Cell motility; Cell proliferation; Cellular metabolic process; Immune response; Proteolysis	NA	NA	+ *
Fc receptor-like B (FCRLB; Q6BAA4)	Immune response	+	NA	NA
Fibroblast growth factor-binding protein 1 (FGF-BP1; Q14512)	Cell proliferation; Cellular response to stress;	++ *	-- *	-- *
Folate receptor alpha (FR-alpha; P15328)	Cell differentiation; Cell motility; Cellular metabolic process; Cellular response to stress	--- *	-- *	++ *
Folate receptor gamma (FR-gamma; P41439)		NA	NA	NA
Furin (FURIN; P09958)	Cell motility; Cell proliferation; Cellular metabolic process; Extracellular matrix organization; Proteolysis	--- *	+	+++ *
Galectin-1 (Gal-1; P09382)	Apoptotic process; Cell adhesion; Cell differentiation; Immune response	++ *	-- *	-- *
Glypican-1 (GPC1; P35052)	Cell differentiation; Cellular metabolic process; Chemotaxis	NA	NA	NA
Granzyme B (GZMB; P10144)	Apoptotic process; Immune response; Proteolysis	-	NA	NA
Granzyme H (GZMH; P20718)	Apoptotic process; Immune response; Proteolysis	ND	ND	ND
Hepatocyte growth factor (HGF; P14210)	Angiogenesis; Apoptotic process; Cell differentiation; Cell motility; Cell proliferation; Cellular metabolic process; Cellular response to stress; Chemotaxis; MAPK cascade; Proteolysis	-	NA	NA
ICOS ligand (ICOSLG; O75144)	Cell adhesion; Cell proliferation; Immune response	NA	-	NA
Insulin-like growth factor 1 receptor (IGF1R; P08069)	Apoptotic process; Cell motility; Cell proliferation; Cellular metabolic process; Cellular response to stress; Immune response; MAPK cascade	---	---	-- *
Integrin alpha-V (ITGAV; P06756)	Angiogenesis; Apoptotic process; Cell adhesion; Cell differentiation; Cell motility; Cell proliferation; Cellular metabolic process; Chemotaxis; Extracellular matrix organization; MAPK cascade	--- *	--- *	+ *
Integrin beta-5 (ITGB5; P18084)	Cell adhesion; Cell differentiation; Extracellular matrix organization	--- *	--- *	- *
Interferon gamma receptor 1 (IFN-gamma-R1; P15260)	Immune response	NA	NA	++ *
Interleukin-6 (IL6; P05231)	Angiogenesis; Apoptotic process; Cell adhesion; Cell differentiation; Cell motility; Cell proliferation; Cellular metabolic process; Cellular response to stress; Chemotaxis; Immune response; MAPK cascade; Proteolysis	NA	NA	--- *
Kallikrein-11 (hK11; Q9UBX7)		++ *	+	-- *
Kallikrein-13 (KLK13; Q9UKR3)	Proteolysis	+	+	-
Kallikrein-14 (hK14; Q9P0G3)	Proteolysis	+	+ *	+
Kallikrein-8 (hK8; O60259)	Cell differentiation; Cell proliferation; Cellular response to stress	++ *	+ *	-
Kit ligand (SCF; P21583)	Apoptotic process; Cell adhesion; Cell differentiation; Cell motility; Cell proliferation; Cellular metabolic process; MAPK cascade	-- *	-- *	++ *
Ly6/PLAUR domain-containing protein 3 (LYPD3; O95274)	Cell adhesion	NA	NA	NA
Melanoma-derived growth regulatory protein (MIA; Q16674)	Cell proliferation	+	-	-
Mesothelin (MSLN; Q13421)	Cell adhesion	ND	ND	ND
Methionine aminopeptidase 2 (MetAP 2; P50579)	Cellular metabolic process; Proteolysis	NA	NA	+
MHC class I polypeptide-related sequence A and B (MIC-A/B; Q29983,Q29980)	Cell adhesion; Cellular response to stress; Immune response	NA	NA	--- *
Midkine (MK; P21741)	Apoptotic process; Cell differentiation; Cell motility; Cellular metabolic process	NA	NA	++ *
Mothers against decapentaplegic homolog 5 (MAD homolog 5; Q99717)	Cell differentiation; Cellular metabolic process	NA	NA	+
Mucin-16 (MUC-16; Q8WXI7)	Cell adhesion; Cellular metabolic process;	NA	NA	+
Nectin-4 (PVRL4; Q96NY8)	Cell adhesion	ND	ND	ND
Pancreatic prohormone (PPY; P01298)	Cellular response to stress	ND	ND	ND
Podocalyxin (PODXL; O00592)	Cell adhesion; Cell differentiation; Cell motility	NA	+	NA
Pro-epidermal growth factor (EGF; P01133)	Angiogenesis; Cell motility; Cell proliferation; Cellular metabolic process; MAPK cascade; Proteolysis	-- *	-	++ *
Protein CYR61 (CYR61; O00622)	Angiogenesis; Apoptotic process; Cell adhesion; Cell differentiation; Cell motility; Cell proliferation; Cellular metabolic process; Chemotaxis; Extracellular matrix organization; MAPK cascade; Proteolysis	-- *	-- *	-- *
Protein S100-A11 (S100A11; P31949)	Cell proliferation; Cellular metabolic process	--- *	--- *	+
Protein S100-A4 (S100A4; P26447)	Cell differentiation	++ *	+++ *	+
Proto-oncogene tyrosine-protein kinase receptor Ret (RET; P07949)	Apoptotic process; Cell adhesion; Cell differentiation; Cell motility; Cellular metabolic process; MAPK cascade; Proteolysis	NA	NA	NA
Protransforming growth factor alpha (TGF-alpha; P01135)	Cell proliferation; Cellular metabolic process; MAPK cascade	+++ *	+++ *	+
Receptor tyrosine-protein kinase erbB-2 (ERBB2; P04626)	Angiogenesis	--- *	--- *	+ *
Receptor tyrosine-protein kinase erbB-3 (ERBB3; P21860)	Apoptotic process; Cell adhesion; Cell differentiation; Cell motility; Cell proliferation; Cellular metabolic process; MAPK cascade	---	--	++ *
Receptor tyrosine-protein kinase erbB-4 (ERBB4; Q15303)	Apoptotic process; Cell differentiation; Cell motility; Cell proliferation; Cellular metabolic process; MAPK cascade	+	-- *	-- *
R-spondin-3 (RSPO3; Q9BXY4)	Angiogenesis	++ *	++	-
Secretory carrier-associated membrane protein 3 (Secretory carrier membrane protein 3) (SCAMP3; O14828)		--- *	--- *	-
Seizure 6-like protein (SEZ6L; Q9BYH1)		ND	ND	ND
SPARC (SPARC; P09486)	Angiogenesis; Cell motility; Cell proliferation; Extracellular matrix organization;	-- *	-- *	-
Syndecan-1 (SYND1; P18827)	Cell differentiation; Cell motility; Cellular metabolic process	NA	NA	++ *
T-cell leukemia/lymphoma protein 1A (TCL1A; P56279)		ND	ND	ND
TGF-beta receptor type-2 (TGFR-2; P37173)	Angiogenesis; Apoptotic process; Cell adhesion; Cell differentiation; Cell motility; Cell proliferation; Cellular metabolic process	-- *	-- *	+ *
Tissue factor pathway inhibitor 2 (TFPI-2; P48307)		---- *	---- *	-- *
T-lymphocyte surface antigen Ly-9 (LY9; Q9HBG7)	Cell adhesion; Cell differentiation; Immune response	+	-	-
Toll-like receptor 3 (TLR3; O15455)	Apoptotic process; Cell differentiation; Cellular metabolic process; Cellular response to stress; Immune response; MAPK cascade	-- *	--- *	-- *
Transmembrane glycoprotein NMB (GPNMB; Q14956)	Cell adhesion; Cell differentiation; Immune response	+	+	-
Tumor necrosis factor ligand superfamily member 10 (TRAIL; P50591)	Apoptotic process; Cellular metabolic process; Immune response; Proteolysis	-- *	-- *	-- *
Tumor necrosis factor ligand superfamily member 13 (TNFSF13; O75888)	Cell proliferation; Cellular metabolic process	ND	ND	ND
Tumor necrosis factor ligand superfamily member 6 (FASLG; P48023)	Angiogenesis; Apoptotic process; Cell differentiation; Cell proliferation; Cellular metabolic process; Immune response; Proteolysis	ND	ND	ND
Tumor necrosis factor receptor superfamily member 19 (TNFRSF19; Q9NS68)	Apoptotic process; Cellular metabolic process; Cellular response to stress; MAPK cascade	-- *	- *	++ *
Tumor necrosis factor receptor superfamily member 4 (TNFRSF4; P43489)	Apoptotic process; Cell adhesion; Cell proliferation; Cellular metabolic process; Immune response; MAPK cascade	NA	+	NA
Tumor necrosis factor receptor superfamily member 6B (TNFRSF6B; O95407)	Apoptotic process; Cell proliferation; Cellular metabolic process; Immune response; MAPK cascade	NA	NA	NA
Tyrosine-protein kinase ABL1 (ABL1; P00519)	Apoptotic process; Cell adhesion; Cell differentiation; Cell motility; Cell proliferation; Cellular metabolic process; Cellular response to stress; Extracellular matrix organization; Immune response; MAPK cascade	----	----	-
Tyrosine-protein kinase Lyn (LYN; P07948)	Apoptotic process; Cell adhesion; Cell differentiation; Cell motility; Cell proliferation; Cellular metabolic process; Cellular response to stress; Chemotaxis; Immune response; MAPK cascade	-- *	-- *	-
Vascular endothelial growth factor A (VEGFA; P15692)	Angiogenesis; Apoptotic process; Cell adhesion; Cell differentiation; Cell motility; Cell proliferation; Cellular metabolic process; Cellular response to stress; Chemotaxis; MAPK cascade; Proteolysis; Response to hypoxia	--- *	---- *	-- *
Vascular endothelial growth factor receptor 2 (VEGFR-2; P35968)	Angiogenesis; Apoptotic process; Cell adhesion; Cell differentiation; Cell motility; Cell proliferation; Cellular metabolic process; Cellular response to stress; Chemotaxis; Extracellular matrix organization; MAPK cascade	ND	ND	ND
Vascular endothelial growth factor receptor 3 (VEGFR-3; P35916)	Angiogenesis; Apoptotic process; Cell motility; Cell proliferation; Cellular metabolic process; Cellular response to stress; MAPK cascade	ND	ND	ND
VEGF coregulated chemokine 1 (CXL17; Q6UXB2)	Angiogenesis; Cell differentiation; Chemotaxis	+	NA	NA
Vimentin (VIM; P08670)	Cell differentiation	-- *	-- *	+ *
WAP four-disulfide core domain protein 2 (WFDC2; Q14508)	Proteolysis	+	+	-
Wnt inhibitory factor 1 (WIF-1; Q9Y5W5)	Cell differentiation	-- *	NA	NA
WNT1-inducible-signaling pathway protein 1 (WISP-1; O95388)	Cell adhesion	NA	+	NA
Xaa-Pro aminopeptidase 2 (XPNPEP2; O43895)		+	-	-

^†^ information gathered from reference [29]; ND: protein not detected in any of the median samples; NA: protein not detected in any of median samples for one or more cell lines; Difference in normalized protein expression between −12–−8 (----), −8–−4 (---), −4–−1 (--), −1–0 (-), 0–1 (+), 1–4 (++), 4–8 (+++), negative difference means the first cell line has lower expression than the second (1st vs. 2nd) and vice versa; * statistically different protein expression (*p* < 0.05).

**Table 4:** Summary of previously published IC_50_ values of doxorubicin in different liver cancer cell lines. Values are shown as IC_50_ (µM).

**Cell Lines**	**Experimental Conditions**					
Incubation	24 h	24 h	36 h	48 h	72 h	48 h
Reference	[33]	[67]	[35]	[34]	[68]	[69]
**Cell line**	**IC_50_ (µM ± St. dev)**					
HepaRG	1.73 ± 0.38	-	-	-	-	
HepG2	2.33 ± 0.05	-	3.85 ± 0.59	1.91 (1.52–2.4)	0.029 ± 0.002	0.288 (0.25–0.32)
HepG2.2.15	3.15 ± 0.92	-	-	-	-	
HLE	-	-	-	-	0.67 ± 0.06	
HLF	-	-	-	-	0.76 ± 0.04	
HT-17	-	-	-	-	6.0 ± 1.9	
Huh-7	2.55 ± 0.10	-	0.47 ± 0.9	3.38 (2.57–4.46)	0.37 ± 0.01	0.36 (0.29–0.42)
Li-7	-	-	-	-	0.46 ± 0.01	
PLC/DOR	-	48.63 ± 17.04	-	-	-	
PLC/PRF/5	-	0.93 ± 0.29	-	-	-	
PLC/PRF/6	-	-	-	-	1.2 ± 0.04	
sk-Hep1	-	-	-	-	0.031 ± 0.002	
SNU449	-	-	-	24.86 (17.97–34.40)	-	1.30 (0.84–0.90)

## Supplementary Materials

The following are available online at https://www.mdpi.com/2072-6694/11/7/1024/s1, Figure S1: Measurement of HIF_1_α and PDK_1_ on three liver cancer cell lines (HepG_2_, Huh7 and SNU_449_) cultured in normoxic and hypoxic conditions, Figure S2: Normalized protein concentrations for each analyzed biomarker and all analyzed conditions and samples, Figure S3: Seeding density and cell viability after 24 h.
