# Supplementary material for: Liver Cancer Cell Lines Treated with Doxorubicin under Normoxia and Hypoxia: Cell Viability and Oncologic Protein Profile
Source: Cancers (Basel). 2019 Jul 20;11(7):1024. doi: 10.3390/cancers11071024 (PMC6678640; doi:10.3390/cancers11071024)

Supplementary information for: **Liver cancer cell lines treated with doxorubicin and hypoxia: cell viability and oncologic protein profile**

By Ilse R. Dubbelboer, Natasa Pavlovic, Femke Heindryckx, Erik Sjögren, and Hans Lennernäs

Figure S1. Measurement of HIF1α and PDK1 on three liver cancer cell lines (HepG2, Huh7 and SNU449) cultured in normoxic and hypoxic conditions. Hypoxia was generated by adding CoCl2 to the cell-culturing medium.

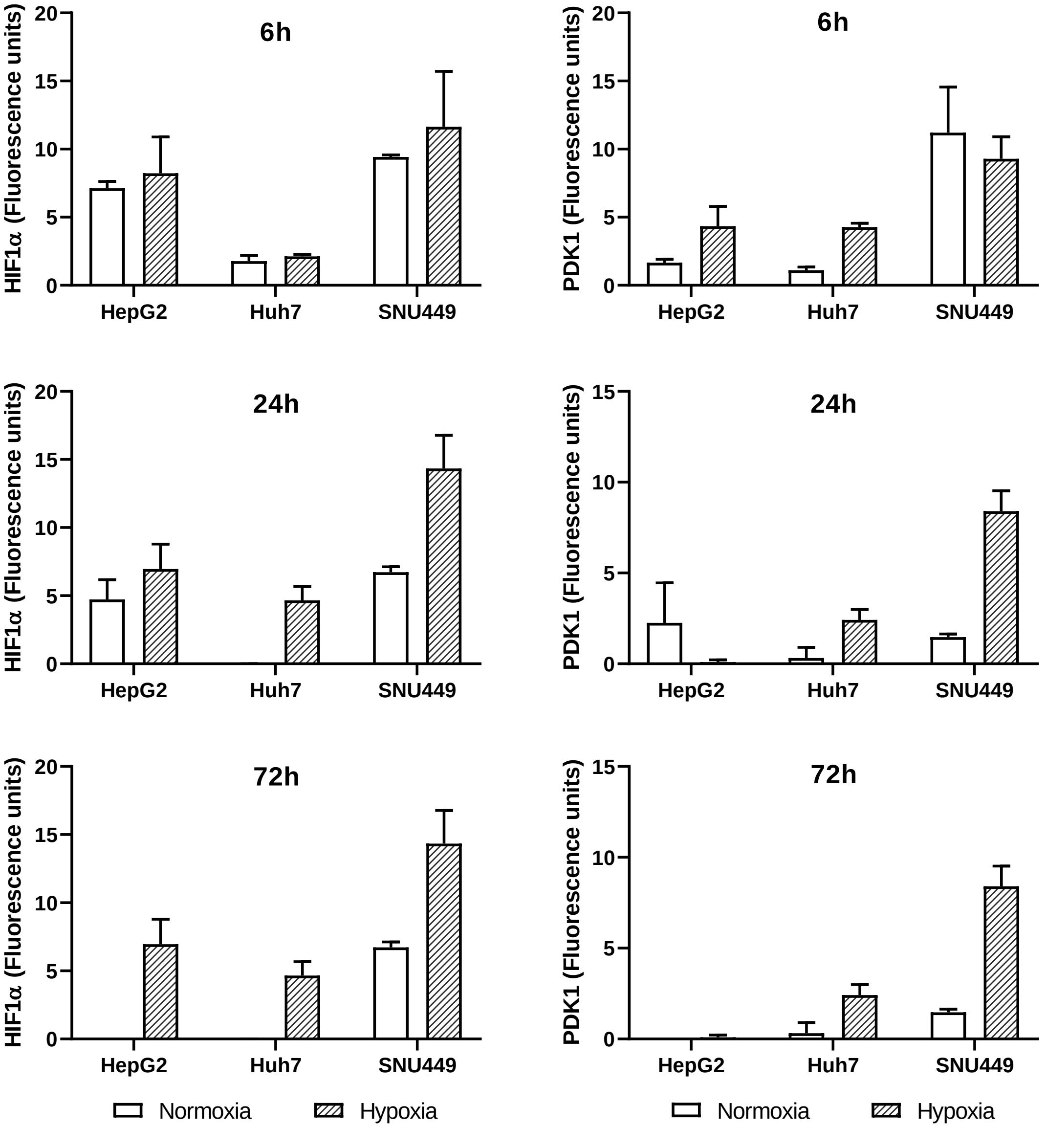

Supplementary information for: **Liver cancer cell lines treated with doxorubicin and hypoxia: cell viability and oncologic protein profile**
By Ilse R. Dubbelboer, Natasa Pavlovic, Femke Heindryckx, Erik Sjögren, and Hans Lennernäs

Figure S2. Normalized protein concentrations for each analyzed biomarker and all analyzed conditions and samples. This is the raw data, i.e. no processed data.

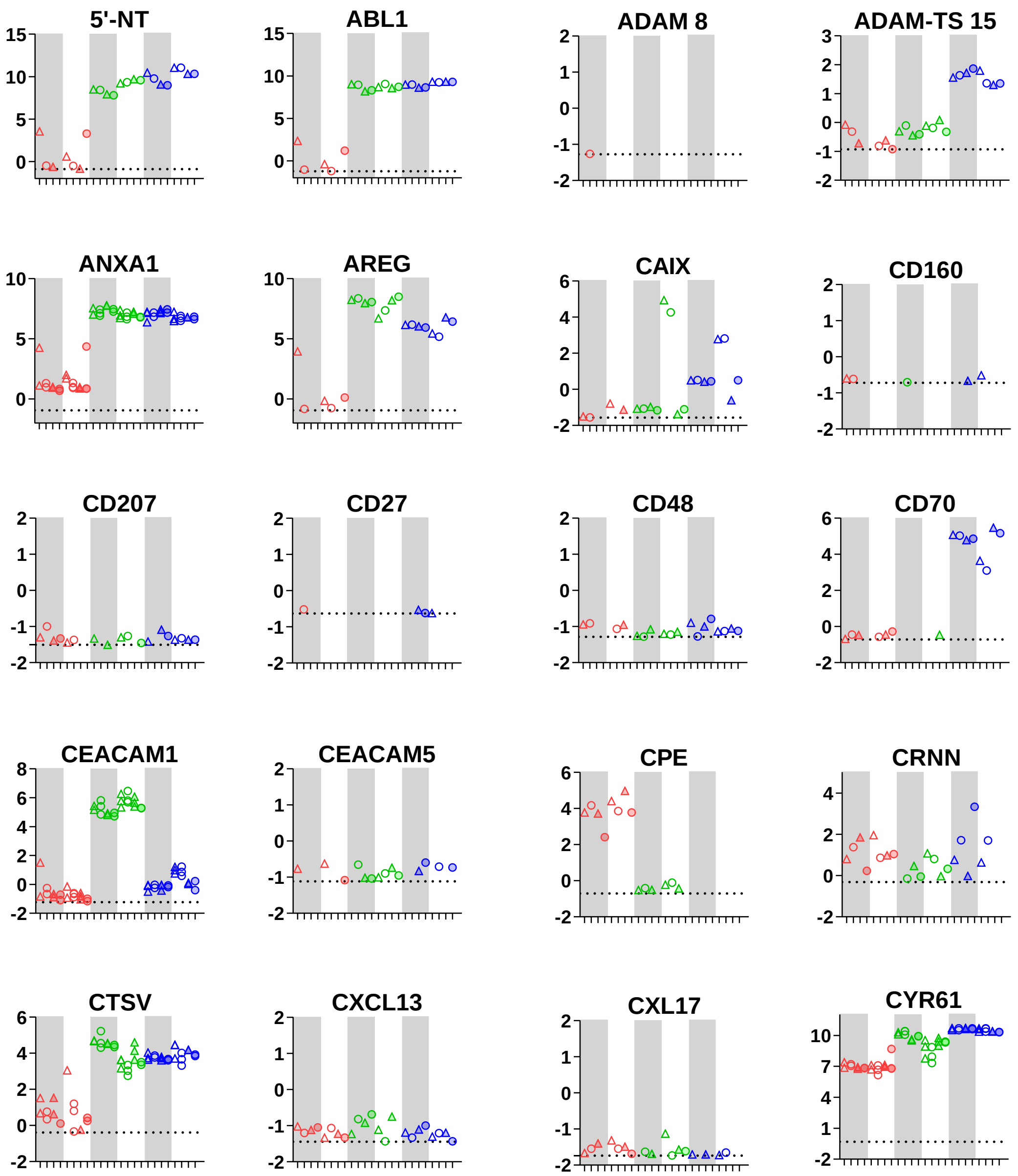

Doxorubicin exposure: Triangles: 0.1μM, Circles: 0μM
Oxygen conditions: Filled symbols: Normoxia, Open symbols: Hypoxia
Exposure time: Grey lines: 6h, White lines: 72h
Cell lines: HepG2, Huh7, SNU449
Horizontal line: LOQ of analysis method

Supplementary information for: **Liver cancer cell lines treated with doxorubicin and hypoxia: cell viability and oncologic protein profile**
By Ilse R. Dubbelboer, Natasa Pavlovic, Femke Heindryckx, Erik Sjögren, and Hans Lennernäs

Continuation Figure S2

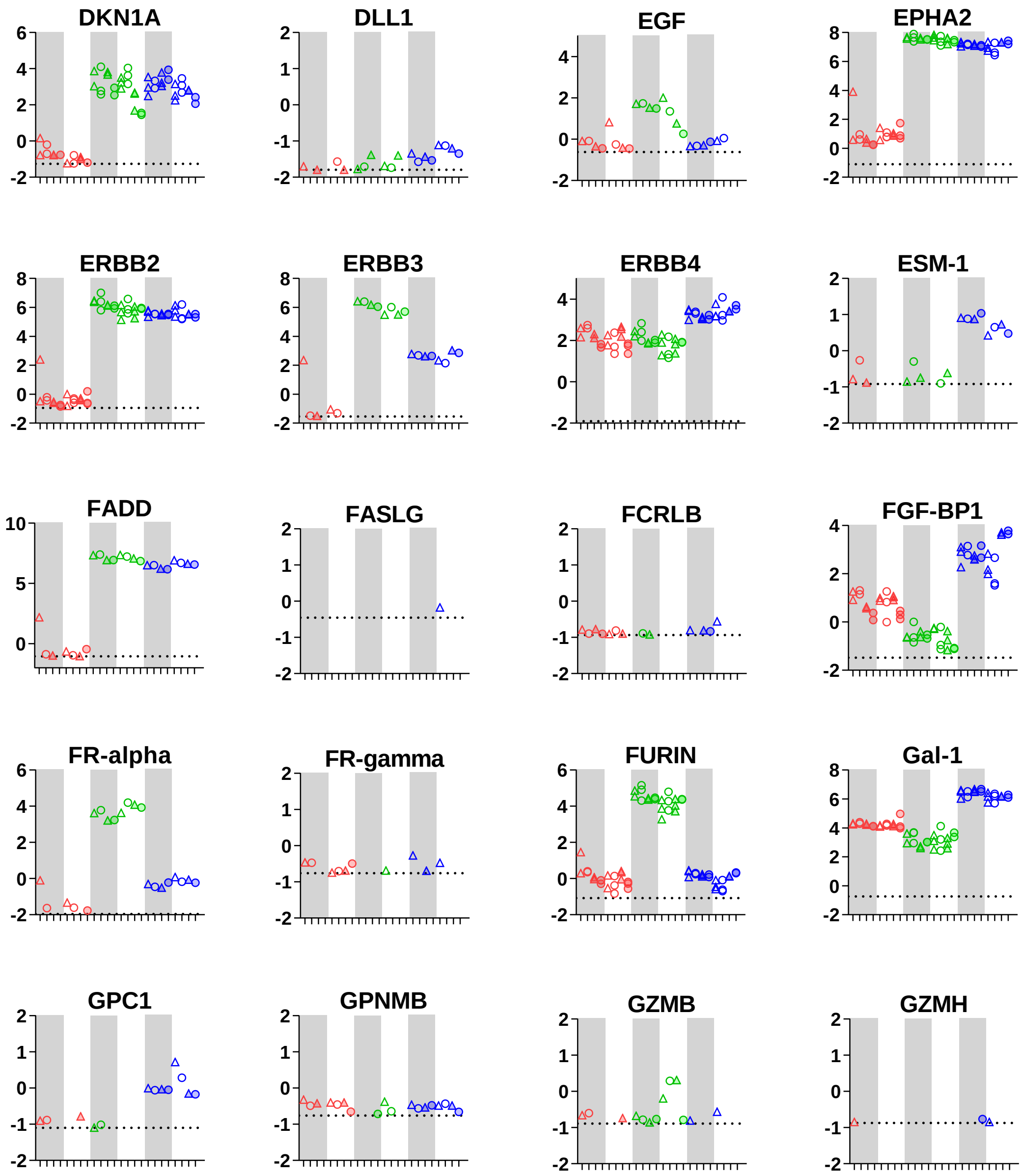

Doxorubicin exposure: Triangles: 0.1µM, Circles: 0µM
Oxygen conditions: Filled symbols: Normoxia, Open symbols: Hypoxia
Exposure time: Grey lines: 6h, White lines: 72h
Cell lines: HepG2, Huh7, SNU449
Horizontal line: LOQ of analysis method

Supplementary information for: **Liver cancer cell lines treated with doxorubicin and hypoxia: cell viability and oncologic protein profile**

By Ilse R. Dubbelboer, Natasa Pavlovic, Femke Heindryckx, Erik Sjögren, and Hans Lennernäs

Continuation Figure S2

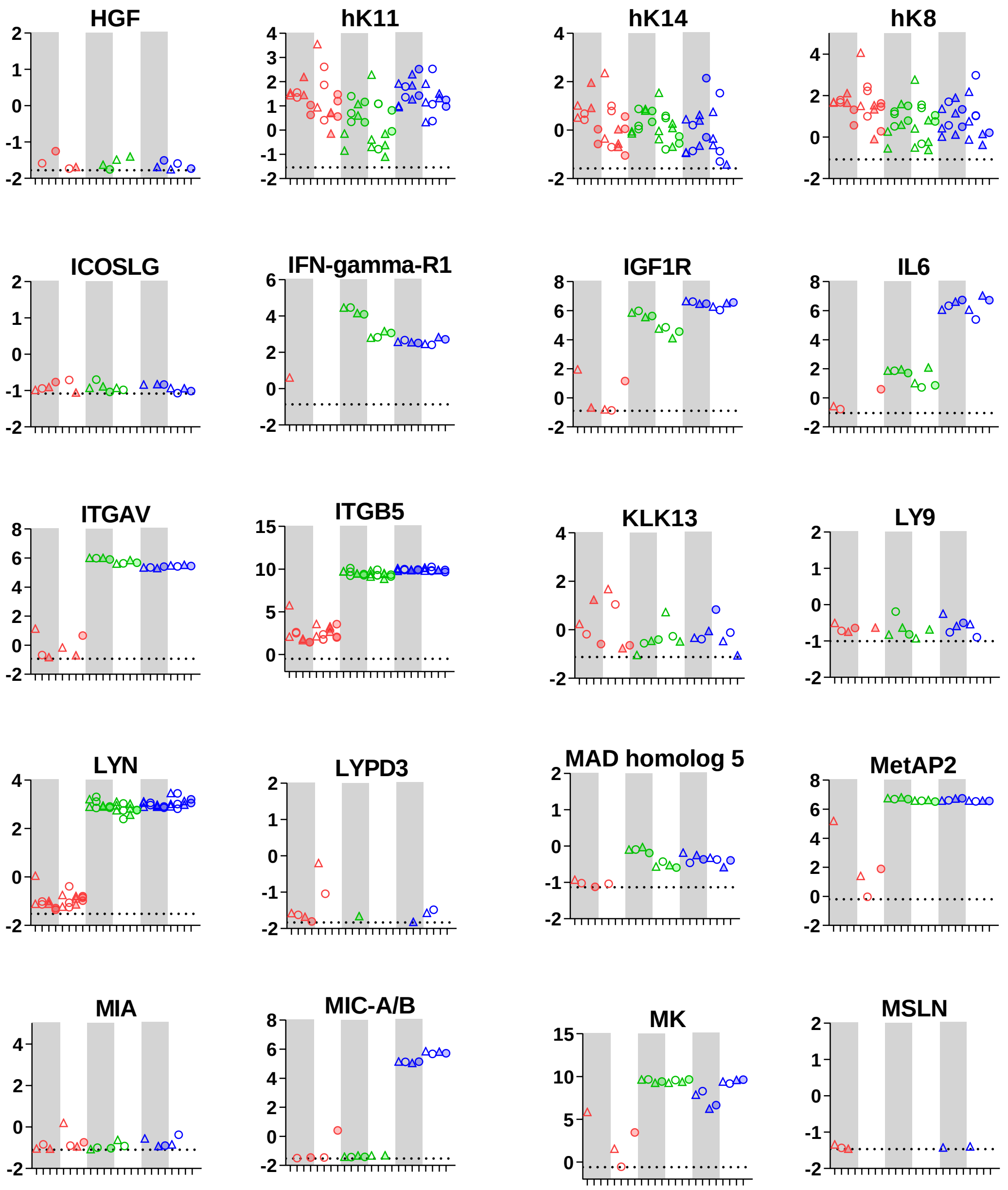

Doxorubicin exposure: Triangles: 0.1µM, Circles: 0µM
Oxygen conditions: Filled symbols: Normoxia, Open symbols: Hypoxia
Exposure time: Grey lines: 6h, White lines: 72h
Cell lines: HepG2, Huh7, SNU449
Horizontal line: LOQ of analysis method

Supplementary information for: **Liver cancer cell lines treated with doxorubicin and hypoxia: cell viability and oncologic protein profile**

By Ilse R. Dubbelboer, Natasa Pavlovic, Femke Heindryckx, Erik Sjögren, and Hans Lennernäs

Continuation Figure S2

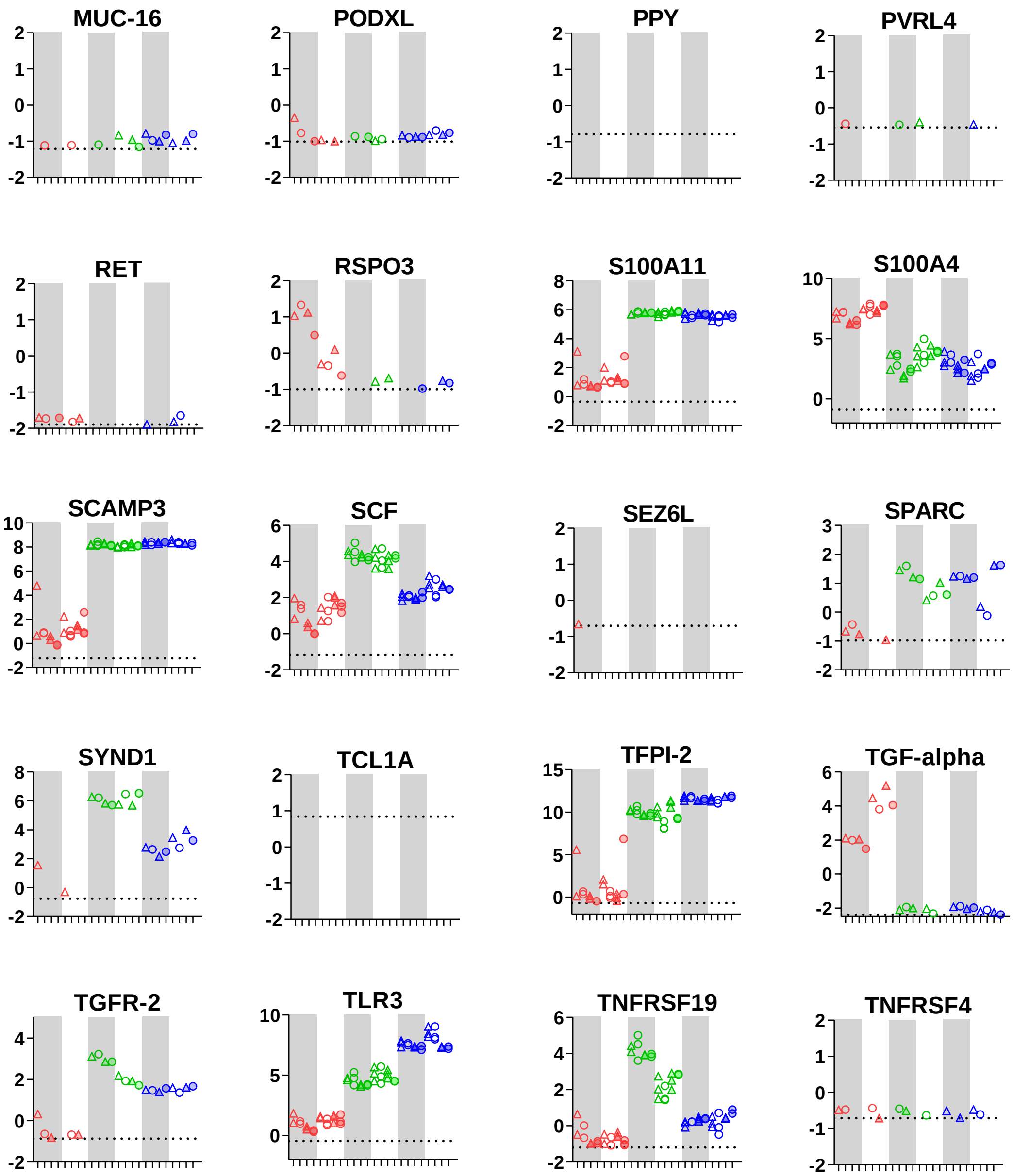

Doxorubicin exposure: Triangles: 0.1µM, Circles: 0µM
Oxygen conditions: Filled symbols: Normoxia, Open symbols: Hypoxia
Exposure time: Grey lines: 6h, White lines: 72h
Cell lines: HepG2, Huh7, SNU449
Horizontal line: LOQ of analysis method

Supplementary information for: **Liver cancer cell lines treated with doxorubicin and hypoxia: cell viability and oncologic protein profile**
By Ilse R. Dubbelboer, Natasa Pavlovic, Femke Heindryckx, Erik Sjögren, and Hans Lennernäs

Continuation Figure S2

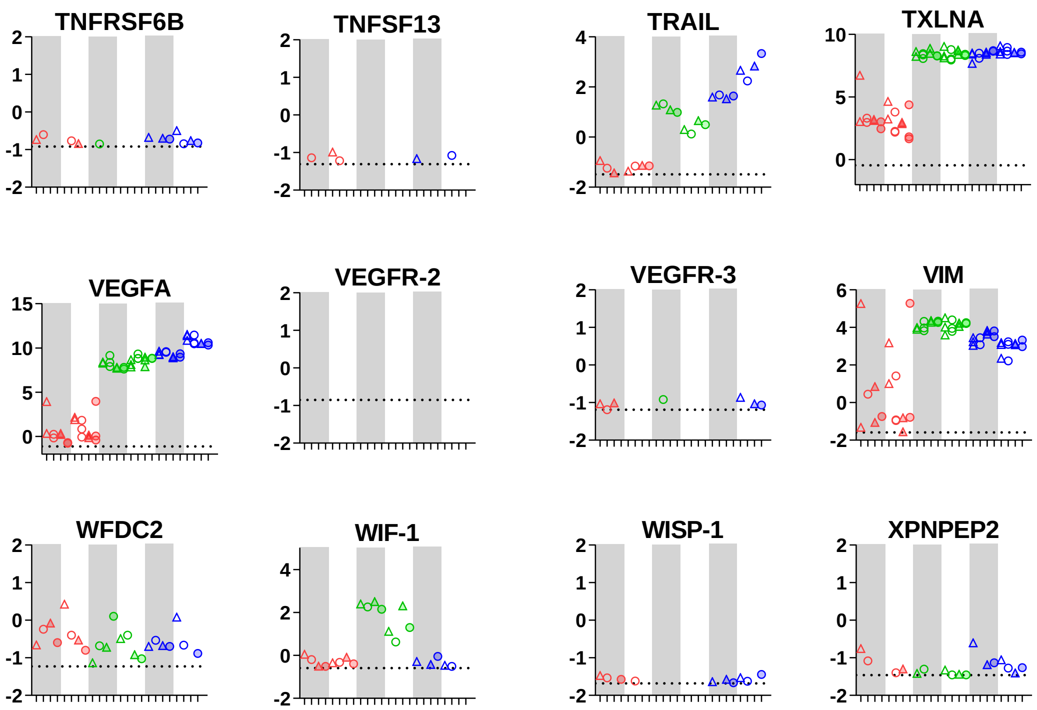

Doxorubicin exposure: Triangles: 0.1µM, Circles: 0µM
Oxygen conditions: Filled symbols: Normoxia, Open symbols: Hypoxia
Exposure time: Grey lines: 6h, White lines: 72h
Cell lines: HepG2, Huh7, SNU449
Horizontal dotted line: LOQ of analysis method

Supplementary information for: **Liver cancer cell lines treated with doxorubicin and hypoxia: cell viability and oncologic protein profile**

By Ilse R. Dubbelboer, Natasa Pavlovic, Femke Heindryckx, Erik Sjögren, and Hans Lennernäs

Figure S3. Seeding density and cell viability after 24h. Cell viability was measured with a resazurin reduction assay.

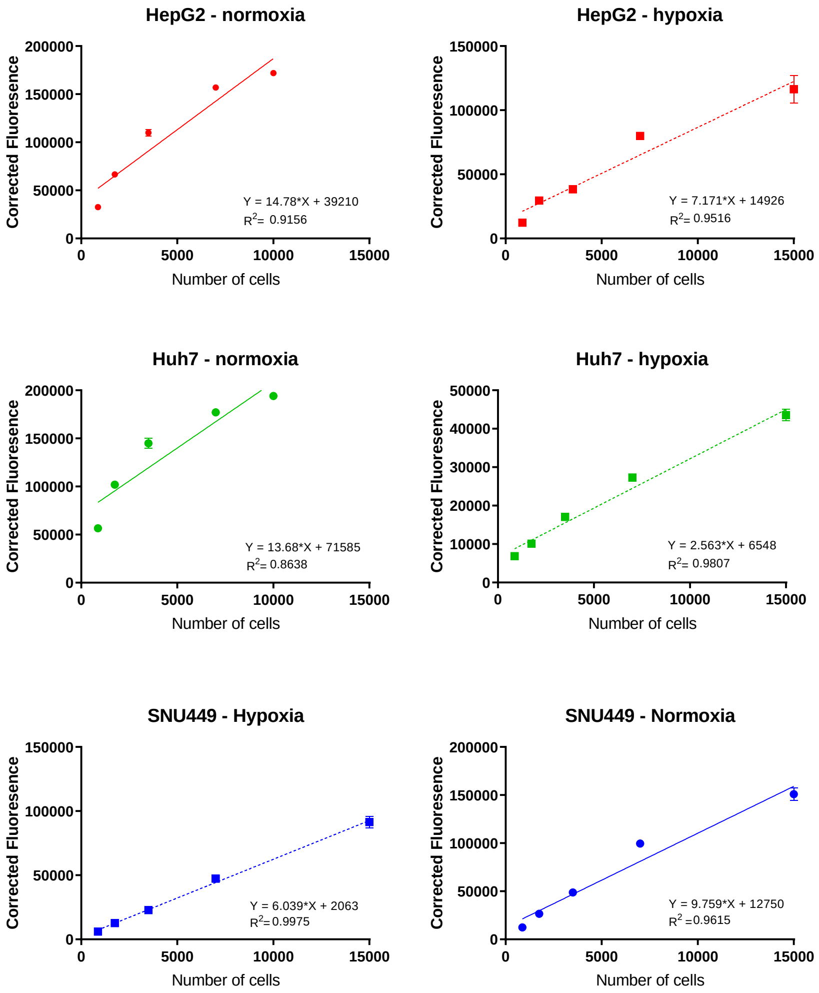

Supplement: Supplementary file 1 [file cancers-11-01024-s001.pdf]
